# Development and validation of an interpretable prehospital return of spontaneous circulation (P-ROSC) score for patients with out-of-hospital cardiac arrest using machine learning: A retrospective study

**DOI:** 10.1016/j.eclinm.2022.101422

**Published:** 2022-05-06

**Authors:** Nan Liu, Mingxuan Liu, Xinru Chen, Yilin Ning, Jin Wee Lee, Fahad Javaid Siddiqui, Seyed Ehsan Saffari, Andrew Fu Wah Ho, Sang Do Shin, Matthew Huei-Ming Ma, Hideharu Tanaka, Marcus Eng Hock Ong, PAROS Clinical Research Network Investigators

**Affiliations:** a Duke-NUS Medical School, National University of Singapore, 8 College Road, Singapore 169857, Singapore; b Health Services Research Centre, Singapore Health Services, Singapore, Singapore; c SingHealth AI Health Program, Singapore Health Services, Singapore, Singapore; d SingHealth Duke-NUS Global Health Institute, Singapore, Singapore; e Institute of Data Science, National University of Singapore, Singapore, Singapore; f National Neuroscience Institute, Singapore, Singapore; g Department of Emergency Medicine, Singapore General Hospital, Singapore, Singapore; h Department of Emergency Medicine, School of Medicine, Seoul National University, Seoul, Republic of Korea; i Department of Emergency Medicine, National Taiwan University Hospital, National Taiwan University, Taipei, Taiwan; j Graduate School of Emergency Medical System, Kokushikan University, Tokyo, Japan

**Keywords:** Out-of-hospital cardiac arrest, Return of spontaneous circulation, Interpretable machine learning, Score

## Abstract

**Background:**

Return of spontaneous circulation (ROSC) before arrival at the emergency department is an early indicator of successful resuscitation in out-of-hospital cardiac arrest (OHCA). Several ROSC prediction scores have been developed with European cohorts, with unclear applicability in Asian settings. We aimed to develop an interpretable prehospital ROSC (P-ROSC) score for ROSC prediction based on patients with OHCA in Asia.

**Methods:**

This retrospective study examined patients who suffered from OHCA between Jan 1, 2009 and Jun 17, 2018 using data recorded in the Pan-Asian Resuscitation Outcomes Study (PAROS) registry. AutoScore, an interpretable machine learning framework, was used to develop P-ROSC. On the same cohort, the P-ROSC was compared with two clinical scores, the RACA and the UB-ROSC. The predictive power was evaluated using the area under the curve (AUC) in the receiver operating characteristic analysis.

**Findings:**

170,678 cases were included, of which 14,104 (8.26%) attained prehospital ROSC. The P-ROSC score identified a new variable, prehospital drug administration, which was not included in the RACA score or the UB-ROSC score. Using only five variables, the P-ROSC score achieved an AUC of 0.806 (95% confidence interval [CI] 0.799–0.814), outperforming both RACA and UB-ROSC with AUCs of 0.773 (95% CI 0.765–0.782) and 0.728 (95% CI 0.718–0.738), respectively.

**Interpretation:**

The P-ROSC score is a practical and easily interpreted tool for predicting the probability of prehospital ROSC.

**Funding:**

This research received funding from SingHealth Duke-NUS ACP Programme Funding (15/FY2020/P2/06-A79).

**Research in context**

**Evidence before the study**

We searched PubMed without language constraints for studies about ROSC prediction in OHCA patients published between Jan 1, 2003 and Jan 31, 2022. We used terms ("prediction") AND ("return of spontaneous circulation" OR "ROSC") AND ("out-of-hospital cardiac arrest" OR "OHCA"). Various models for estimating the likelihood of gaining ROSC were developed and validated primarily on European cohorts. The discriminative ability of these models for Asian patients may be limited because of considerable differences in individual characteristics and EMS systems. To our knowledge, the P-ROSC score generated in this study is the first score to estimate prehospital ROSC probability for patients with OHCA in Asian settings.

**Added value of the study**

Our study developed the point-based P-ROSC score based on one of the largest OHCA cohorts. This score was derived using a novel scoring strategy that exploited the local characteristics of each community and produced an integrated score through weighting. In doing so, the disparate data distributions among different communities could be addressed. Besides, the readily accessible variables used in P-ROSC score make it easy to understand, interpret, and apply. Moreover, compared with previous scores, P-ROSC demonstrated superior discrimination performance.

**Implications of all the available evidence**

The parsimonious P-ROSC score developed in our study is a potentially helpful tool for the prediction of prehospital ROSC tailored to the Asian population.

Alt-text: Unlabelled box

## Introduction

Out-of-hospital cardiac arrest (OHCA) significantly burdens the healthcare system and society. It has been estimated that 53.1 incidences of emergency medical services (EMS)-treated OHCA happen per 100,000 persons per year in North America, 34.4 in Europe, and 59.4 in Asia.^1^^,^^2^ Despite efforts by the medical community and the public, OHCA remains a leading cause of mortality worldwide, with patient outcomes varying significantly. The estimated survival to discharge rate, weighted by person-years, was 6.8% in North America, 7.6% in Europe, and 3% in Asia.^2^ This variation requires thorough investigation for contributing factors and highlights the possibility of considerable improvement in patient outcomes.

The relationship between preclinical and clinical variables and the outcomes of OHCA have been studied extensively.^3^, ^4^, ^5^, ^6^ In four aspects, these variables affect the 'chain of survival': patient-related (e.g., age, gender, comorbidity), event-related (e.g., location of arrest, time of arrest, witness status, bystander cardiopulmonary resuscitation [CPR], automated external defibrillator [AED] at the scene, arrest etiology, type of heart rhythm, agony breathing), system-related (e.g., time to CPR, time to defibrillation, dispatcher-assisted CPR, characteristics, and quality of EMS system), and therapy (e.g., use of advanced life support).^7^ While survival and favorable neurological outcomes have been extensively investigated,^8^ the return of spontaneous circulation (ROSC) also gained attention as an early indicator of resuscitation success. Additionally, ROSC as an outcome measure allows for focused investigation of prehospital factors.

Among the various models that predict the likelihood of gaining ROSC, the ROSC after Cardiac Arrest (RACA) score developed with the German Resuscitation Registry is the most well validated. RACA calculates the probability of ROSC (at scene or en route to hospital) by using variables available before arrival at the emergency department (ED).^5^ The RACA score was not designed to be used as a prediction tool on the spot to facilitate resuscitation decisions. Instead, by providing a predicted ROSC rate, the score has the potential of identifying weak points in the 'chain of survival', enabling comparison of studies conducted in different communities and cohorts, serving as a 'quality indicator' of varying resuscitation strategies and EMS systems. In two European studies, the RACA score was applied to assess the impact of airway management on ROSC rate,^9^ and to evaluate the effect of resuscitation staffing.^10^

Several studies have attempted to validate the RACA score in their populations, which yielded mixed results.^10^, ^11^, ^12^, ^13^, ^14^ The discrepancies, although partially explained by different inclusion and exclusion criteria and the fluctuating nature of resuscitation practices over time, imply the need for further adjustment of the score for individual communities.^12^ In a recent effort to create an operational score, Baldi et al. generated the Utstein-Based ROSC (UB-ROSC) score by analyzing patient data using Utstein templates to identify the probability of sustaining ROSC and survival to hospital admission of OHCA victims.^1^ UB-ROSC is a relatively new instrument and has not been widely validated with external patient cohorts, particularly in Asia.

The high level of variability and wide array of variables collected in the Pan-Asian Resuscitation Outcomes Study (PAROS) make the registry a valuable dataset for identifying new contributing factors that impact patient outcomes. In light of the significant differences in patient characteristics and EMS systems between Pan-Asian communities and European cohorts, our study attempted to develop an interpretable Prehospital ROSC (P-ROSC) score tailored explicitly for patients with OHCA in Asia. To achieve this, we used AutoScore, a machine learning framework that allows semi-automatic variable selection and predictive modeling.^15^^,^^16^ Moreover, we compared P-ROSC with RACA and UB-ROSC scores on a separate testing dataset to evaluate their performance in prehospital ROSC prediction.

## Methods

### Study design and setting

PAROS is an international clinical research network founded in 2010, which aims to understand OHCA in Asia. The PAROS registry now includes communities in Singapore, Japan, South Korea, Taiwan, Thailand, Malaysia, China, Philippines, Vietnam, Pakistan, India, Lebanon, and the United Arab Emirates (UAE). A comprehensive description of the network has been published previously.^17^^,^^18^ A common taxonomy and case report form have been developed to standardize data collection and recording. The collected variables range from patient-related (e.g., age, gender, etiology of arrest), event-related (e.g., bystander CPR, witnessed status), EMS-related (e.g., response time) to patient outcomes. The PAROS registry contains a mix of urban and rural communities, and their EMS systems vary in terms of organizational structure, staffing, and service capability.^17^^,^^19^

This study was a retrospective analysis of OHCA data collected in the PAROS registry between Jan 1, 2009 and Jun 17, 2018. The reporting of this study followed the guideline of TRIPOD^20^ (Transparent Reporting of a multivariable prediction model for Individual Prognosis Or Diagnosis). OHCA is characterized by the absence of pulse, unresponsiveness, and apnea. ROSC was defined as the return of a palpable pulse (transient or sustained) at the scene or while en route to the ED. Patients who did not receive attempted resuscitation and were immediately pronounced dead in the field were excluded from the analysis. Pediatric cases (age < 18 years), cases without age information, cases not conveyed by EMS, or cases with "Do not resuscitate" orders were also excluded.

In the PAROS registry, the arrest location was not reported in six Japanese cities, and bystander AED information was not recorded in Taiwan data; thus, they were treated as a new category to indicate unknown status. For other variables, information was likely missing at random, allowing us to exclude cases with incomplete information without substantially biasing subsequent analyses. Also excluded from this study were communities with fewer than 1000 cases, as insufficient data would result in unreliable prediction models.

The final selected data was divided into non-overlapping training (70%), validation (10%), and testing (20%) sets, with testing data consisting of the most recent cases from each community. The data split was stratified by ROSC to ensure that the training and validation sets had a similar distribution of data. This study utilized the training and validation sets to develop the P-ROSC score and the testing set to evaluate the P-ROSC, RACA, and UB-ROSC scores.

### P-ROSC score derivation using AutoScore

AutoScore is a framework developed to automate the derivation of risk scores using a combination of machine learning and regression modeling.^15^^,^^21^ The score generated is point-based, making it easy to apply to a variety of clinical settings. At first, candidate variables (age, gender, etiology of arrest, witness status, arrest location, first arrest rhythm, bystander CPR, bystander AED, response time, whether call was during the day, prehospital drug administration, prehospital defibrillation, and prehospital advanced airway) were pre-selected based on clinical relevance. Besides, the community index was also selected as a location identification. These variables were then fed into the random forest (RF), a widely used machine learning algorithm, to rank their importance in outcome prediction.^22^^,^^23^

Next, variables were transformed to ensure clinical applicability. The categorical variables were re-grouped for purpose of analysis. In particular, for the first rhythm, ventricular fibrillation (VF), ventricular tachycardia (VT), and unknown shockable were grouped into shockable rhythm; while pulseless electrical activity (PEA), asystole, and unknown unshockable were combined into unshockable rhythm. In the case of continuous variables, the values were classified into categories, with cut-off values determined by the quantiles of the data points.^15^ Subsequently, multivariable logistic regression was used to build models and produce scores. A parsimony plot was generated based on RF-ranked variables, in which model performance (as measured by the area under the curve [AUC]) was plotted against the number of variables in each model. If a variable was ranked at the tail of the RF-based ranking, or if adding this variable could not improve performance according to the parsimony plot, it might be considered to have low predictive ability. The optimal number of variables was determined by balancing the predictive ability with model complexity, and the cut-off values for each variable were fine-tuned through an iterative process based on clinical domain knowledge and intermediate predictive performance on the validation set.^15^

To derive the score, we considered data heterogeneity as sample size, event rate, and patient characteristics varied by community. After determining the variables and corresponding categories in the previous step, scores were developed by analyzing training and validation data from each community. Subsequently, for each category of a variable, its corresponding score point was computed as a weighted average across all communities. For each community *i*, a weightage $w_{i}$ was formulated as $w_{i}=(\sqrt{\left(AUC_{i}\right)}\times N_{i}^{3})/(\sum_{i=1}^{M}\sqrt{\left(AUC_{i}\right)}\times N_{i}^{3})\times 100\%$ where $N_{i}$ was the training sample size, $AUC_{i}$ was the AUC value obtained based on the validation set, and M was the total number of communities. It was assumed that a community-specific score with higher discrimination power or a score derived from a larger training sample could contribute more to the aggregated score. Lastly, we followed the AutoScore pipeline to normalize the score breakdowns and create the P-ROSC score with a maximum value of 100.^15^

Moreover, the P-ROSC score was converted into a probability using weighted logistic regression on the validation set to enhance the interpretability. We produced a conversion table to display score cut-offs and their corresponding prediction performance. Physicians could choose a cut-off tailored to their applications based on the likelihood of a prehospital ROSC and metrics such as sensitivity and specificity.

Considering the risk of losing information due to the prior recoding work for categorical variables and categorization for continuous variables in the AutoScore, we conducted a sensitivity analysis where we built a multivariable logistic regression model using the same variables as the P-ROSC score, but without any recoding and categorization. This model was tested on the same cohort as the P-ROSC score.

### RACA score calculation

The RACA score was calculated using the original formula developed by Gräsner et al.^5^ The probability of ROSC was linked to the linear predictor (X) by the logit function, where X was a linear combination of patient-related variables (age, gender, and cardiac arrest etiology), event-related variables (location of cardiac arrest, witnessed status, and bystander CPR), and EMS-related variables (first rhythm and response time). Due to differences in variable categories between the PAROS registry and the German Resuscitation Registry, we re-grouped the variables in accordance with our previous study.^12^

### UB-ROSC score calculation

The UB-ROSC score was calculated using the scoring table reported in Baldi et al.^1^ The probability of ROSC could be computed by applying a random effect model to a linear combination of seven variables, including age, gender, etiology, arrest location, witness with bystander CPR, rhythm and response time. The PAROS registry variables were recoded accordingly to fit for the calculation of UB-ROSC. The re-grouping for the etiology was the same as for RACA. For arrest location, unknown status was considered as home; industrial area was treated as workplace; transportation center was regarded as a public building. First rhythm was re-grouped into shockable and un-shockable, while the arrest witnessed was categorized as no, witnessed, and EMS witnessed. Arrest witnessed and bystander CPR were then fused into a 5-category feature with levels: EMS witnessed, not witnessed and no CPR, not witnessed and yes CPR, witnessed and no CPR, and witnessed and yes CPR.

### Statistical analysis

In the descriptive analysis of variables of interest, continuous variables were summarized by mean and standard deviation as well as median and interquartile range, while categorical variables were summarized by frequency and percentage. Comparisons between groups (outcome vs non-outcome) were performed using the Chi-square test for categorical variables and the Mann-Whitney U test for continuous variables after the Kolmogorov-Smirnov test verified non-normality.

The P-ROSC, RACA, and UB-ROSC scores were assessed using the receiver operating characteristic (ROC) analysis, where the AUC values with 95% confidence intervals (CIs) were reported.^24^ To further evaluate the differences in discrimination, DeLong's test was conducted to pair wisely compare the AUC values. The data analysis and model building were performed using R version 4.0.2 (The R Foundation for Statistical Computing), mainly based on packages “AutoScore”,^15^^,^^21^ “tableone”^25^ and “pROC”.^26^ A two-sided *p* < 0.05 was considered statistically significant.

The local Institutional Review Boards approved the study. The waiver of informed consent was approved for the collection of data.

### Role of the funding source

The funders were not involved in the study design, collection, analysis, and interpretation of data, nor did they have a role in the writing of the paper and decision to submit the paper for publication. All authors had access to the data in the study and had final responsibility for the decision to submit for publication.

## Results

### Baseline characteristics of study cohort

**Figure 1:**
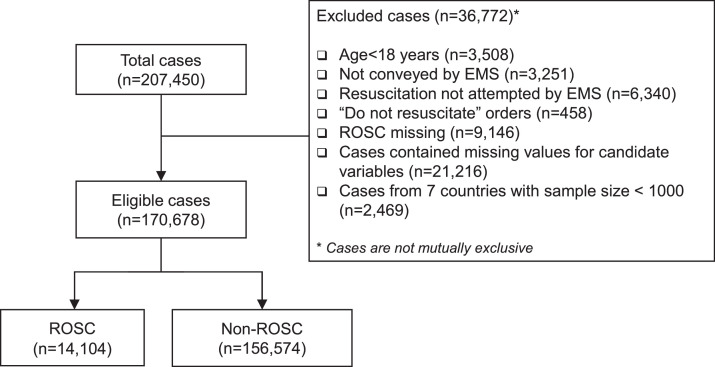
Flow of data selection from the Pan-Asian Resuscitation Outcomes Study (PAROS) registry. EMS: emergency medical services. ROSC: return of spontaneous circulation.

Between Jan 1, 2009 and Jun 17, 2018, 207,450 patients who suffered from OHCAs were recorded in the PAROS database. Among them, 3,508 (1.69%) were pediatric, 3,251 (1.57%) were not conveyed by EMS, 6,340 (3.06%) were pronounced dead in the field, 458 (0.22%) had a “do-not-resuscitate” order, and 9,146 (4.41%) had missing outcomes. These cases were excluded from data analysis. Among all candidate variables, the largest missing rate was 59.6% (arrest location), and the second-largest missing rate was 19.8% (bystander AED); the missingness in these two variables was considered a new category. Except for these two variables, the largest missing rate was 6.5% (first rhythm), and missing rates for other variables were less than 3%. As a result, 21,216 (10.23%) cases with missing values were excluded. Furthermore, cases (2,469, 1.19%) from the Philippines, Vietnam, Pakistan, UAE, Thailand, Malaysia, and China were excluded due to the small sample size. Of the remaining 170,678 cases, 130,370 were from Japan, 14,388 from South Korea, 12,067 from Taiwan, and 13,853 from Singapore. 14,104 (8.26%) patients achieved prehospital ROSC. The flow of cohort formation is displayed in Figure 1.

Baseline characteristics of patients with OHCA in training, validation, and testing datasets are described in Table 1 and those for overall eligible PAROS registry are presented in the supplementary Table S1. As observed in the entire cohort, ROSC was significantly associated with younger age (71 years vs 76 years, median age; 68.96 years vs 72.22 years, mean age) and the response time was significantly different between ROSC and non-ROSC groups (6.74 min vs 7.06 min, mean time). Patients with ROSC were more likely to be male (65.8% vs 58.5%), have witnessed arrests by professionals (13.8% vs 6.4%) or laypersons (63.0% vs 33.8%) and have shockable first rhythm (32.4% vs 7.1%). ROSC was significantly associated with the prehospital drug administration (33.4% vs 10.9%).

**Table 1:** Descriptive summary of variables in the P-ROSC score on out-of-hospital cardiac arrest (OHCA) cases.

	Training set				Validation set				Test set (20% latest cases)			
	Overall (*N* = 119477)	ROSC (*N* = 9628)	Non-ROSC (*N* = 109849)	*p*-value	Overall (*N* = 17067)	ROSC (*N* = 1375)	Non-ROSC (*N* = 15692)	*p*-value	Overall (*N* = 34134)	ROSC (*N* = 3101)	Non-ROSC (*N* = 31033)	*p*-value
**Age (median [IQR / mean (SD))**	76 [62, 84] / 71.77 (16.89)	71 [60, 81] / 68.76 (16.38)	76 [62, 84] / 72.04 (16.91)	<0.001	76 [62, 84] / 71.73 (16.79)	71 [60, 81] / 68.92 (16.10)	76 [62, 84] / 71.97 (16.82)	<0.001	76 [64, 85] / 72.64 (16.69)	72 [60, 82] / 69.46 (16.38)	77 [64, 85] / 72.96 (16.68)	<0.001
**Time to EMS arrival (min) (median [IQR] / mean (SD))**	6 [5, 8] / 6.99 (9.99)	6 [5, 8] / 6.66 (7.68)	6 [5, 8] / 7.02 (10.17)	<0.001	6 [5, 8] / 6.90 (3.29)	6 [5, 8] / 6.64 (3.27)	6 [5, 8] / 6.93 (3.29)	<0.001	7 [5, 8] / 7.25 (10.70)	7 [5, 8] / 7.03 (3.03)	6.45 [5, 8] / 7.27 (11.18)	0.053
**First rhythm (n (%))**				<0.001				<0.001				<0.001
Shockable	11096 (9.3)	3101 (32.2)	7995 (7.3)		1603 (9.4)	466 (33.9)	1137 (7.2)		2994 (8.8)	1004 (32.4)	1990 (6.4)	
Unshockable	108381 (90.7)	6527 (67.8)	101854 (92.7)		15464 (90.6)	909 (66.1)	14555 (92.8)		31140 (91.2)	2097 (67.5)	29043 (93.6)	
**Witnessed (n (%))**				<0.001				<0.001				<0.001
No	68264 (57.1)	2288 (23.8)	65976 (60.1)		9688 (56.8)	285 (20.7)	9403 (59.9)		18929 (55.5)	702 (22.6)	18227 (58.7)	
Professional	8510 (7.1)	1341 (13.9)	7169 (6.5)		1264 (7.4)	211 (15.3)	1053 (6.7)		2217 (6.5)	393 (12.7)	1824 (5.9)	
Lay person	42703 (35.7)	5999 (62.3)	36704 (33.4)		6115 (35.8)	879 (63.9)	5236 (33.4)		12988 (38.1)	2006 (64.7)	10982 (35.4)	
**Prehospital Drug (n (%))**				<0.001				<0.001				<0.001
Yes	14649 (12.3)	3066 (31.8)	11583 (10.5)		2014 (11.8)	415 (30.2)	1599 (10.2)		5159 (15.1)	1226 (39.5)	3933 (12.7)	
No	104828 (87.7)	6527 (67.8)	98266 (89.5)		15053 (88.2)	960 (69.8)	14093 (89.8)		28975 (84.9)	1875 (60.5)	27100 (87.3)	

EMS: emergency medical services.

PEA: pulseless electrical activity.

ROSC: return of spontaneous circulation.

IQR: interquartile range.

SD: standard deviation.

*p*-value: the *p*-value of Mann-Whitney U test or Chi-square test.

### P-ROSC score development

**Figure 2:**
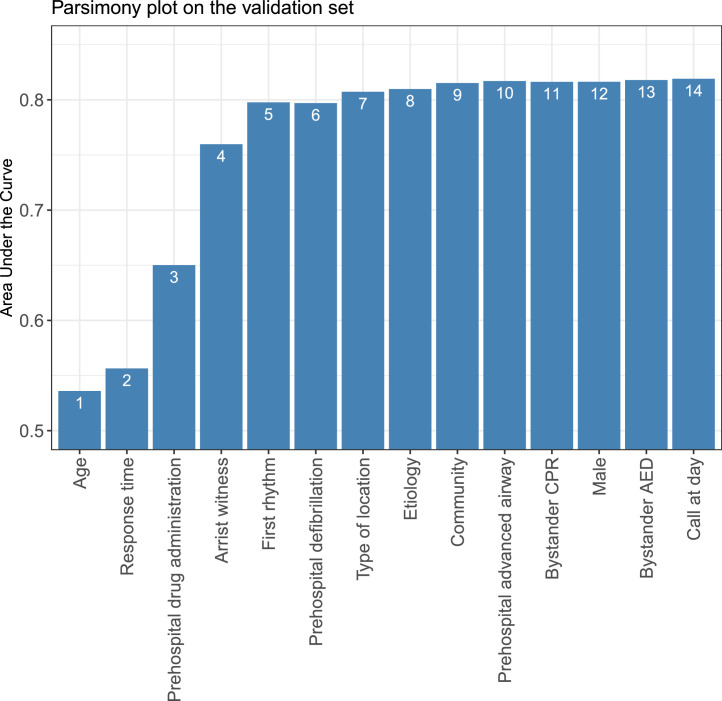
Parsimony plot showing model performance (area under the curve) against model complexity (number of variables) during model selection in the P-ROSC score development when considering datasets from different communities as a whole.

To select a subset of variables for model development, all candidate variables were ranked by importance, and a parsimony plot was generated (Figure 2). As a result of achieving a tradeoff between model performance and complexity, the top five variables were included in the final model because of their high predictive ability: age, response time, first rhythm, witnessed status, and prehospital drug administration. By adjusting for coefficients and fine-tuning the models, we obtained the scoring tables for each community (Supplementary Table S2). The final P-ROSC score had a range of 0–100 (Table 2). Younger age, shorter time to EMS arrival, and prehospital drug administration contributed to an increased likelihood of gaining prehospital ROSC. The presence of a professional witness raised ROSC probability more than a lay witness. The shockable first rhythm was the most significant predictor of ROSC, as it alone had a score of 30. For a specific threshold, the probability of prehospital ROSC and the accompanying metrics were recorded in Table 3.

**Table 2:** The score table of the P-ROSC score.

Variable	Score
**Age (year)**	
<60	13
60-85	10
85-90	7
>=90	0
**Time to EMS arrival (minute)**	
<5	9
5-9	7
9-12	3
>=12	0
**First rhythm**	
Non-shockable	0
Shockable	30
**Arrest witnessed**	
No	0
Professional	27
Lay person	19
**Prehospital drug administration**	
Yes	21
No	0

Interval ($q_{1}$-$q_{2}$) represents $q_{1}\leq x<q_{2}$.

EMS: emergency medical services.

**Table 3:** Conversion table of the P-ROSC score.

P-ROSC score cut-off (≥)	Predicted ROSC probability (%)	Percentage of patients with ROSC (%)	Sensitivity (95% CI)	Specificity (95% CI)
10	17.9	91	98.8% (98.3–99.3%)	9.7% (9.2–10.1%)
20	26.3	51	89.9% (88.3–91.3%)	52.8% (52.1–53.6%)
30	39.7	43	84.9% (83–86.7%)	60.3% (59.5–61.1%)
40	54.9	20	58.3% (55.9–60.9%)	83.1% (82.5–83.7%)
50	69.2	13	47.2% (44.6–49.9%)	90.2% (89.7–90.6%)
60	80.6	8	33% (30.5–35.5%)	93.8% (93.5–94.2%)
70	88.5	2	9.3% (7.8–10.8%)	98.2% (98.0–98.4%)
80	94.1	2	4.4% (3.3–5.5%)	98.8% (98.6–98.9%)
90	96.3	1	1.2% (0.7–1.8%)	99.5% (99.4–99.6%)

ROSC: probability of return of spontaneous circulation.

CI: confidence interval.

### Performance evaluation

**Figure 3:**
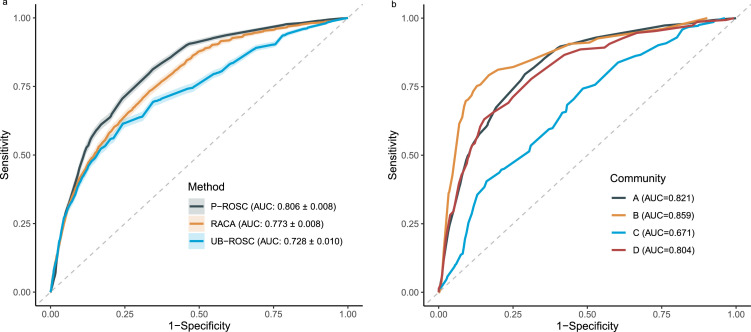
Receiver operating characteristic (ROC) curve of the RACA score, UB-ROSC score and P-ROSC score on out-of-hospital cardiac arrest (OHCA) cases in the Pan-Asian Resuscitation Outcomes Study (PAROS) from (a) all 4 communities and (b) individual communities. In (a), area under the curve (AUC) value for each score is presented in the legend. In (b), AUC value for each community is presented in the legend.

Using the same testing dataset, we compared the prediction performance among P-ROSC, RACA, and UB-ROSC scores (Figure 3a). The RACA score achieved an AUC of 0.773 (95% CI: 0.765–0.782), and the UB-ROSC score yielded an AUC of 0.728 (95% CI: 0.718–0.738). Overall, the P-ROSC score displayed the highest discrimination power with an AUC of 0.806 (95% CI: 0.799–0.814). Furthermore, a pairwise discrimination comparison of these three scores was presented in Table 4, which demonstrated that P-ROSC significantly outperformed the other two scores. Figure 3b illustrates the performance of P-ROSC in four individual communities, in which the score revealed excellent results in three of them. The P-ROSC score correlated with the observed outcome, where the average score was 64.3 for patients with ROSC and 37.1 for those without ROSC.

**Table 4:** Pairwise discrimination comparison of AUC values of the P-ROSC, RACA, and UB-ROSC scores.

	P-ROSC		RACA		UB-ROSC	
Method	Diff.	*p*-value	Diff.	*p*-value	Diff.	*p*-value
P-ROSC	-	-	-	-	-	-
RACA	0.033	<0.001	-	-	-	-
UB-ROSC	0.078	<0.001	0.045	<0.001	-	-

Diff: AUC difference.

*p*-value: the *p*-value of AUC difference between two models.

In the sensitivity analysis, without recoding and categorization, the multivariable logistic regression model with the same variables as the P-ROSC score yielded a lower AUC of 0.715 (95% CI: 0.706–0.724). This result demonstrated that the recoding for categorical variables and categorization for continuous variables could lead to better predictive performance.

## Discussion

In this registry-based study, we developed the P-ROSC score, an interpretable instrument that predicts the chances of achieving ROSC before arrival at the ED. Based on a large multinational dataset, P-ROSC is the first score to estimate the ROSC probability of patients with OHCA in Asia. The parsimonious P-ROSC score exhibited excellent discrimination capabilities, presenting it as a potentially helpful tool to aid clinical decision-making.

The P-ROSC score is easily accessible. It has only five well-defined variables, all of which are readily available during prehospital resuscitation. Four variables (age, witnessed status, response time, and first rhythm) are shared with RACA^5^ and UB-ROSC^1^ scores, suggesting their significance in predicting ROSC. Moreover, a variable common to both RACA and UB-ROSC scores – the arrest location – is absent from the P-ROSC score. It is important to note that the various categories of arrest location may vary concerning cultural or geographic factors, and for this reason, its inclusion may limit the generalizability of the score in different settings. On the other hand, prehospital drug administration is selected as a predictor in P-ROSC while omitted from RACA and UB-ROSC. This reinforces the critical role of prehospital medication in determining favorable outcomes such as ROSC and survival.^27^^,^^28^

For risk scores such as RACA, the logit function is required to translate a linear combination of predictors into a probability of ROSC.^5^^,^^10^^,^^12^ In contrast, P-ROSC is a simple additive score computed by accumulating the individual scores of each variable, which allows quick calculation and transparent interpretation.^16^ To be more specific, the point-based scoring structure illustrates the relative importance of categories within and between variables, where the relative importance corresponds to clinical intuition.^15^ Table 2 shows, for example, that the shockable first rhythm has a score of 30, making it the single most important factor in estimating the probability of ROSC.

In developing P-ROSC, we adopted a divide-and-combine approach to account for the population differences among communities. Japanese data has the highest rate of ROSC, almost twice the rate in Taiwan data; Singapore data shows a high prevalence of prehospital drug administration, while the corresponding number in South Korea's data is much lower. The disparity in the dataset was addressed by creating four individual risk scores for each community and combining them into a single P-ROSC score using a weighting mechanism. This could provide the score with good adaptability in a variety of settings. Nevertheless, the method cannot handle data from countries with small sample sizes since a robust predictive model is required for each community.^29^ Techniques such as federated learning^30^ or swarm learning^31^ may help reduce the requirement for a large sample size.

In addition to its predictive value, the P-ROSC score may also suggest areas for prioritization and improvement, especially when resources are scarce. For instance, because P-ROSC includes response time as a variable, we speculate that optimizing ambulance dispatch^32^ to reduce waiting time would be crucial in helping gain prehospital ROSC. However, we need to take caution while interpreting these hypotheses, as EMS is a complex, multifactorial system where survival and favorable neurologic outcomes are even more significant measures to take into account.^33^ Along with the above functions, P-ROSC could also serve as a quality management tool, similar to the RACA score, to provide objective comparisons across EMS systems.^5^

This study has limitations. First, trauma arrests were not included in the P-ROSC, and thus it cannot be generalized to trauma-induced OHCA. Second, recoding predictive variables to align with RACA and UB-ROSC variables might lead to less accurate score calculation. Third, the ROSC definitions in P-ROSC, RACA, and UB-ROSC were different, making a direct comparison between these three scores difficult. Upon implementation, it is worth noting that the actual clinical settings differ geographically and culturally. When high-quality data from other Asian communities becomes available, further external validation of P-ROSC will be conducted. Similar to the validation study conducted by Caputo et al.,^11^ we will consider an interaction test to evaluate the generalizability of the P-ROSC score. Fourth, as a multinational study, the caveat of system variation across countries may compromise the reliability of certain variables, such as time. A further investigation should be conducted to determine whether the community's time clocked by ambulance, EMS, and ED differed. Lastly, specific medical histories were excluded from score derivation due to a sizeable missing rate, although they may have contributed to prediction.

We developed the P-ROSC score, a readily accessible risk prediction tool for ROSC probability estimation. Compared to the RACA and UB-ROSC scores, P-ROSC is more parsimonious with just five variables. It presents the best performance in an Asian cohort consisting of data from Japan, South Korea, Taiwan, and Singapore. Moreover, P-ROSC is interpretable, making it easy to implement and comprehend in busy prehospital settings.

## Data sharing

All data are stored in a secure server environment hosted by Singapore Clinical Research Institute and can be accessed by researchers in the Pan-Asian Resuscitation Outcomes Study (PAROS) Clinical Research Network. For further information, please contact Dr Nan Liu (liu.nan@duke-nus.edu.sg).

## Funding

This research received funding from SingHealth Duke-NUS ACP Programme Funding (15/FY2020/P2/06-A79).

## Contributors

NL conceived and designed the study. NL, ML, XC, YN, and JWL analysed the data. All authors interpreted the data. NL, ML, XC, and YN drafted the manuscript. All authors critically revised the manuscript for intellectual content. NL supervised the study. All authors had access to and verified the data in the study and had final responsibility for the decision to submit for publication.

## Declaration of interests

MEH Ong reports funding from the ZOLL Medical Corporation for a study involving mechanical cardiopulmonary resuscitation devices; grants from the Laerdal Foundation, Laerdal Medical, and Ramsey Social Justice Foundation for funding of the Pan-Asian Resuscitation Outcomes Study; an advisory relationship with Global Healthcare SG, a commercial entity that manufactures cooling devices; and funding from Laerdal Medical on an observation program to their Community CPR raining Centre Research Program in Norway. MEH Ong has a licensing agreement and a patent filed (Application no: 13/047,348) with ZOLL Medical Corporation for a study titled "Method of predicting acute cardiopulmonary events and survivability of a patient". Nan Liu reports funding from SingHealth Duke-NUS ACP Programme Funding for this study. All other authors have no conflict of interests to declare.

## Appendix Supplementary materials

Image, application 1
