# Supplementary material for: Development and validation of an interpretable prehospital return of spontaneous circulation (P-ROSC) score for patients with out-of-hospital cardiac arrest using machine learning: A retrospective study
Source: eClinicalMedicine. 2022 May 6;48:101422. doi: 10.1016/j.eclinm.2022.101422 (PMC9096672; doi:10.1016/j.eclinm.2022.101422)
Supplement: Supplementary file 1 [file mmc1.pdf]

Appendix

Table S1. Overall summary of variables in the P-ROSC score on out-of-hospital cardiac arrest (OHCA) cases from the Pan-Asian Resuscitation Outcomes Study (PAROS).

	**Overall (N=170678)**	**ROSC (N=14104)**	**Non-ROSC (N=156574)**	**p-value**
**Age (median (IQR) / mean (SD))**	76 [62, 84] / 71.95 (16.84)	71 [59, 81] / 68.96 (16.35)	76 [63, 85] / 72.22 (16.86)	<0.001
**Time to EMS arrival (min) (median (IQR) / mean (SD))**	6 [5, 8] / 7.04 (9.69)	6 [5, 8] / 6.74 (6.58)	6 [5, 8] / 7.06 (9.92)	<0.001
**First rhythm (n (%))**				<0.001
Shockable	15693 (9.2)	4571 (32.4)	11122 (7.1)	
Unshockable	154985 (90.8)	9533 (67.6)	145452 (92.9)	
**Witnessed (n (%))**				<0.001
No	96881 (56.0)	3275 (23.2)	93606 (59.8)	
Professional	11991 (7.0)	1945 (13.8)	10046 (6.4)	
Lay person	61806 (36.2)	8884 (63.0)	52922 (33.8)	
**Prehospital Drug (n (%))**				<0.001
Yes	21822 (12.8)	4707 (33.4)	17115 (10.9)	
No	148856 (87.2)	9397 (66.6)	139459 (89.1)	

EMS: emergency medical services. PEA: pulseless electrical activity. ROSC: return of spontaneous circulation. IQR: interquartile range. SD: standard deviation. p-value: the p-value of Mann-Whitney U test or Chi-square test.

Table S2: Pointwise scoring tables for the four communities and the final scoring table.

	Japan	South Korea	Singapore	Taiwan	Final Score
**Age (years)**					
<60	11	17	22	11	13
60-85	7	12	19	11	10
85-90	7	4	11	7	7
>=90	0	0	0	0	0
**Time to EMS arrival (min)**					
<5	7	12	7	19	9
5-9	7	8	7	11	7
9-12	4	4	0	4	3
>=12	0	0	4	0	0
**First rhythm**					
Non-shockable	0	0	0	0	0
Shockable	26	33	41	30	30
**Arrest witness**					
No	0	0	0	0	0
Professional	30	21	26	22	27
Lay person	26	12	4	11	19
**Prehospital drug administration**					
Yes	26	17	4	19	30
No	0	0	0	0	0

Using the formula provided in Method, the weightages are 0.547 for Japan, 0.207 for South Korea, 0.095 for Singapore and 0.151 for Taiwan. Take "age < 60" as an example, its scoring point was $0.547 \times 11 + 0.207 \times 17 + 0.095 \times 22 + 0.151 \times 11 = 13.28$. The baseline for age is "age > 90" with scoring point 0. After setting ceiling total score of 100 and rounding, the P-ROSC scoring point for "age < 60" became 13.

Pan-Asian Resuscitation Outcomes Study Clinical Research Network Investigators

Participating site investigators: H Tanaka (Graduate School of EMS System, Kokushikan University, Tokyo, Japan); Tagami T (Nippon Medical School Tama Nagayama Hospital, Tokyo, Japan); T Nishiuchi (Department of Acute Medicine, Kindai University Faculty of Medicine, Japan); SD Shin (Department of Emergency Medicine, Seoul National University College of Medicine, Seoul, Republic of Korea); HW Ryoo (Department of Emergency Medicine, Kyungpook National University Hospital, Daegu, Korea); MHM Ma (Department of Emergency Medicine, National Taiwan University Hospital Yunlin Branch, Douliou, Taiwan); PCI Ko (Department of Emergency Medicine, National Taiwan University Hospital, College of Medicine, National Taiwan University, Taipei, Taiwan); CW Kuo (Department of Emergency Medicine, Chang-Gung Memorial Hospital, Linkou, Taoyuan, Taiwan); P Khruenkarnchana (International Medical Services, Bangkok Hospital, Bangkok, Thailand); J Supasaowapak (Rajavithi Hospital, Bangkok, Thailand); KD Wong (Emergency Department, Hospital Pulau Pinang, Penang, Malaysia); NE Doctor (Sengkang General Hospital, Singapore); S Arulanandam (Emergency Medical Services Department, Singapore Civil Defence Force, Singapore); HN Gan (Changi General Hospital, Singapore); BSH Leong (National University Hospital, Singapore); SO Cheah (Urgent Care Clinic International, Singapore); WM Ng (Ng Teng Fong General Hospital, Singapore); DR Mao (Khoo Teck Puat Hospital, Singapore); YY Ng (Tan Tock Seng Hospital, Singapore); LP Tham (KK Women's & Children's Hospital, Singapore); R Rao (GVK Emergency Management and Research Institute, Telangana, India); M Vimal (GVK Emergency Management and Research Institute, Telangana, India); FJ Gaerlan (Southern Philippines Medical Center, Davao, Philippines); W Cai (Zhejiang Provincial People's Hospital, Zhejiang, China); SA Zhou (Zhejiang Provincial People's Hospital, Zhejiang, China); M Khursheed (Emergency Department, National Institute of Cardiovascular Diseases, Karachi, Pakistan), DA Nguyen (Bach Mai Hospital, Hanoi, Vietnam); S AlQahtani (National Ambulance, Abu Dhabi, United Arab Emirates); O Al Sakaf (Department of Medical and Technical Affairs, Dubai Corporation for Ambulance Services, Dubai, United Arab Emirates); AL Blewer (Duke University School of Medicine, North Carolina, United States of America).
